# Real-world experience on intravitreal dexamethasone implant in patients with macular edema scheduled to undergo cataract surgery

**DOI:** 10.1186/s12886-023-03093-y

**Published:** 2023-08-09

**Authors:** Chun-Chieh Lai, Shu-Chun Kuo

**Affiliations:** 1 Department of Ophthalmology, National Cheng Kung University Hospital, College of Medicine, National Cheng Kung University, Tainan, Taiwan; 2 Institute of Clinical Medicine, College of Medicine, National Cheng Kung University, Tainan, Taiwan; 3 Department of Ophthalmology, Chi Mei Medical Center, Tainan, Taiwan; 4 Department of Optometry, Chung Hwa University of Medical Technology, No. 901, Zhonghua Rd, Yongkang District, Tainan City, 710 Taiwan

**Keywords:** Dexamethasone implant, Diabetic macular edema, Sustained release, Cataract, Retinal vein occlusion

## Abstract

**Background:**

Patients with pre-existing macular edema (ME) due to diabetes and retinal vein occlusions (RVO) make up a growing population receiving cataract surgery. Surgery is associated with an increased risk of worsening existing ME due to post-surgical inflammation that can be further exacerbated by pre-existing diabetic retinopathy (DR) and retinal vein occlusion. This study aimed to examine the pre-operative use of intravitreal dexamethasone (DEX) implants in patients with ME undergoing cataract surgery.

**Methods:**

A retrospective study was conducted at National Cheng Kung University Hospital in Taiwan involving 19 eyes of 16 patients with DME or ME associated with RVO. All participants received a DEX implant at baseline and underwent phacoemulsification within 3 months after its insertion. Best-corrected visual acuity (BCVA), intraocular pressure (IOP) and central subfield thickness (CST) were evaluated.

**Results:**

DEX implants reduced the CST from baseline (357.8 μm) to pre-surgery (280.8 μm). This reduction below baseline continued to month 6 post-surgery (319.4 μm). From baseline (16.15 mmHg), the mean IOP initially increased pre-surgery (17.78 mmHg) before returning to the baseline value at month 6 post-surgery (16.15 mmHg). All patients improved their BCVA from logMAR 0.943 on average at baseline to logMAR 0.532 at month 6 post-surgery.

**Conclusions:**

The results of the study suggested that patients with ME could benefit from DEX implants before cataract surgery within 3 months to achieve sufficient postoperative inflammation management and limit ME deterioration. DEX implants did not increase IOP post-surgery and was similar to baseline levels.

**Supplementary Information:**

The online version contains supplementary material available at 10.1186/s12886-023-03093-y.

## Background

Cataract surgery is one of the most common surgeries performed in developed countries [1]. Diabetic patients account for approximately a quarter of these surgeries, given that prevalence of cataracts has been demonstrated to be five times higher in this population [1–3]. In addition, hyperglycemia contributes to greater cell permeability within the eye, resulting in edema within the macula, affecting 22% of diabetic patients. It has been demonstrated that more than a quarter of diabetic patients receiving cataract surgery had diabetic macula edema (DME) [2, 3]. Similarly, given its relationship to age, it is not uncommon for patients with retinal vein occlusions (RVO) to present with cataracts. RVO accounts for 12% of eyes with severe vision loss, and such vision loss is predominantly associated with ME [4]. Therefore, diabetic and RVO patients have a greater risk of combined cataracts and ME. Cataract surgery is associated with an increased risk of worsening existing edema due to post-surgical inflammation that can be further exacerbated by pre-existing diabetic retinopathy (DR) [2, 5].

Corticosteroids’ anti-inflammatory, anti-angiogenic, and anti-permeability properties combat three main pathologic mechanisms involved in ME progression [6]. However, the method of administration has a significant impact on the efficacy of corticosteroids. Corticosteroids can be delivered directly into the eye, bypassing the blood–retinal barrier, resulting in a higher local drug concentration and increased systemic safety. However, with the existing ME therapy options, safely delivering therapeutic medication levels to the posterior part of the eye without the need for recurrent redosing continues to be a challenge.

Dexamethasone (DEX) is a corticosteroid with a 6-fold higher anti-inflammatory action than that of prednisolone or triamcinolone, 25 times that of hydrocortisone, and comparable to fluocinolone acetonide. DEX injection into the vitreous humor has been demonstrated to provide higher drug concentrations with lower toxicity; however, DEX has a short half-life of approximately 4 hours after intravitreal injection, restricting its clinical utility [7–9]. A DEX intravitreal implant is a biodegradable implant that delivers a smaller amount of the glucocorticoid DEX over 4–6 months. It is used to treat adult patients with diabetic ME (DME) who are pseudophakic or who are inadequately sensitive to or ineligible for non-corticosteroid therapeutic interventions, as well as ME caused by RVO, or inflammation of the posterior segment of the eye portraying as noninfectious uveitis [10]. Using implants for controlled delivery of DEX into the vitreous space has also been demonstrated to regulate ME in vitrectomized eyes over many months [11, 12].

Several trials have indicated encouraging outcomes utilizing an intravitreal DEX implant to manage postoperative ME in patients with DME and RVO [10, 13, 14]. Most studies reported on an implant that was used at the same time as the cataract surgery. Panozzo et al. observed that DME recurred in 1 out of 19 patients with DME and cataract one month following surgery, and in 74% of patients at 4–5 months post-surgery [14]. In their retrospective analysis, Malclès et al. demonstrated the use of a DEX implant one month before cataract surgery resulted in no visual impairments in the months following surgery [15]. After a 24-week follow-up, an intravitreal DEX implant at the start of phacoemulsification drastically decreased central macular thickness and enhanced visual acuity in a prospective controlled, randomized interventional pilot experiment [16].

As only a few studies have looked at the safety and efficacy of the cataract surgery combined with DEX implantation in patients with ME associated with diabetes or RVO, the present study aims to examine the pre-operative use of intravitreal DEX implants in patients with ME undergoing cataract surgery.

## Methods

This retrospective study was conducted at National Cheng Kung University Hospital in Taiwan. National Cheng Kung University Hospital, a prominent institution, provided the setting for this study, with Dr. Chun-Chieh Lai serving as the sole cataract surgeon involved. Nineteen eyes (ocular dexter = 14, ocular sinister = 5) of sixteen patients with DME or ME associated with RVO were enrolled in the study (Supplementary Fig. 1). Only patients with ME and cataract who received DEX implant injection and cataract surgery within three months were included in this study. This injection served as the baseline injection of DEX implant in this study and does not necessarily represent the first injection. Patients may have received prior injections for ME. However, all the patients had edema at the time of baseline injection and received cataract surgery within 3 months. Patients with follow-ups of less than six months after cataract surgery, and those with insufficient data, were excluded. The study was conducted in accordance with the Declaration of Helsinki and was approved by the hospital’s institutional review board. All participants gave their written informed permission.

All participants received an intravitreal DEX implant at baseline and underwent cataract surgery (phacoemulsification and posterior chamber intraocular lens implantation) within 3 months after its insertion. The recurrence of ME and the timing of DEX implants were also recorded within six months after cataract surgery. Best-corrected visual acuity logMAR (BCVA logMAR), central subfield thickness (CST) and intraocular pressure (IOP) changes between baseline (Pre-DEX), time of cataract surgery (Post-DEX to surgery) and postoperative months (Post-surgery 1,2,3 and 6 months) were evaluated. Statistical analysis, including paired T-test, was performed, and a p-value of < 0.05 was statistically significant.

## Results

Nineteen eyes of sixteen participants were enrolled in the study (Table 1). The mean age of the participants was 72.5 ± 12.2 years, and 75% were women. Most of the subjects were diagnosed with DME (68.8%), while others had RVO (31.2%). Nearly half (43.8%) of the enrolled subjects presented with proliferative DR, while 18.9% had non-proliferative DR. Hypertension was present in 11 patients (68.9%) and hyperlipidemia in five patients (31.3%). Of the total enrolled subjects, nine participants had undergone focal retinal or panretinal photocoagulation, two participants received intravitreal aflibercept, three received intravitreal ranibizumab, and one participant received intravitreal bevacizumab before baseline injection of DEX implant. The mean number of intravitreal DEX injections received before cataract surgery was 4.2.

**Table 1:** Baseline characteristics of the enrolled subjects

Parameters	Value (± SD)
Age (in years), mean (SD)	72.5 (12.2)
Male, n (%)	4 (25.0)
OD, n (%)	5 (31.3)
Diagnosis, n (%)	
DME	11 (68.8)
RVO	5 (31.2)
DM, n (%)	
Type 1 DM	1 (6.3)
Type 2 DM	11 (68.9)
No DM	2 (12.3)
No reports	2 (12.3)
Mean HbA1c, mean (SD)	6.7 (1.1)
DM retinopathy, n (%)	
Non-proliferative	3 (18.8)
Proliferative	7 (43.8)
Hypertension, n (%)	11 (68.9)
Dyslipidemia, n (%)	5 (31.3)
Glaucoma, n (%)	3 (18.8)
Previous treatment before intravitreal dexamethasone implant, n	
Intravitreal bevacizumab	1
Intravitreal ranibizumab	3
Intravitreal aflibercept	2
Focal or panretinal photocoagulation	9
Previous intravitreal injections of DEX implant before surgery, mean number (SD)	4.2 (2.7)

Eight of these 19 eyes (42.1%) had a recurrence of ME (5 with diabetes and 3 with RVO) during follow-ups, and all received a re-injection of DEX implant. The averaged interval between the re-injection and baseline injection of DEX implant was 122.4 days (range from 68 to 186 days) in these 8 patients (all recurrences happened unilaterally in these patients). Noteworthily, patients with ME associated with RVO seemed to have a higher recurrence rate and the need for re-injection of DEX implants.

**Fig. 1:**
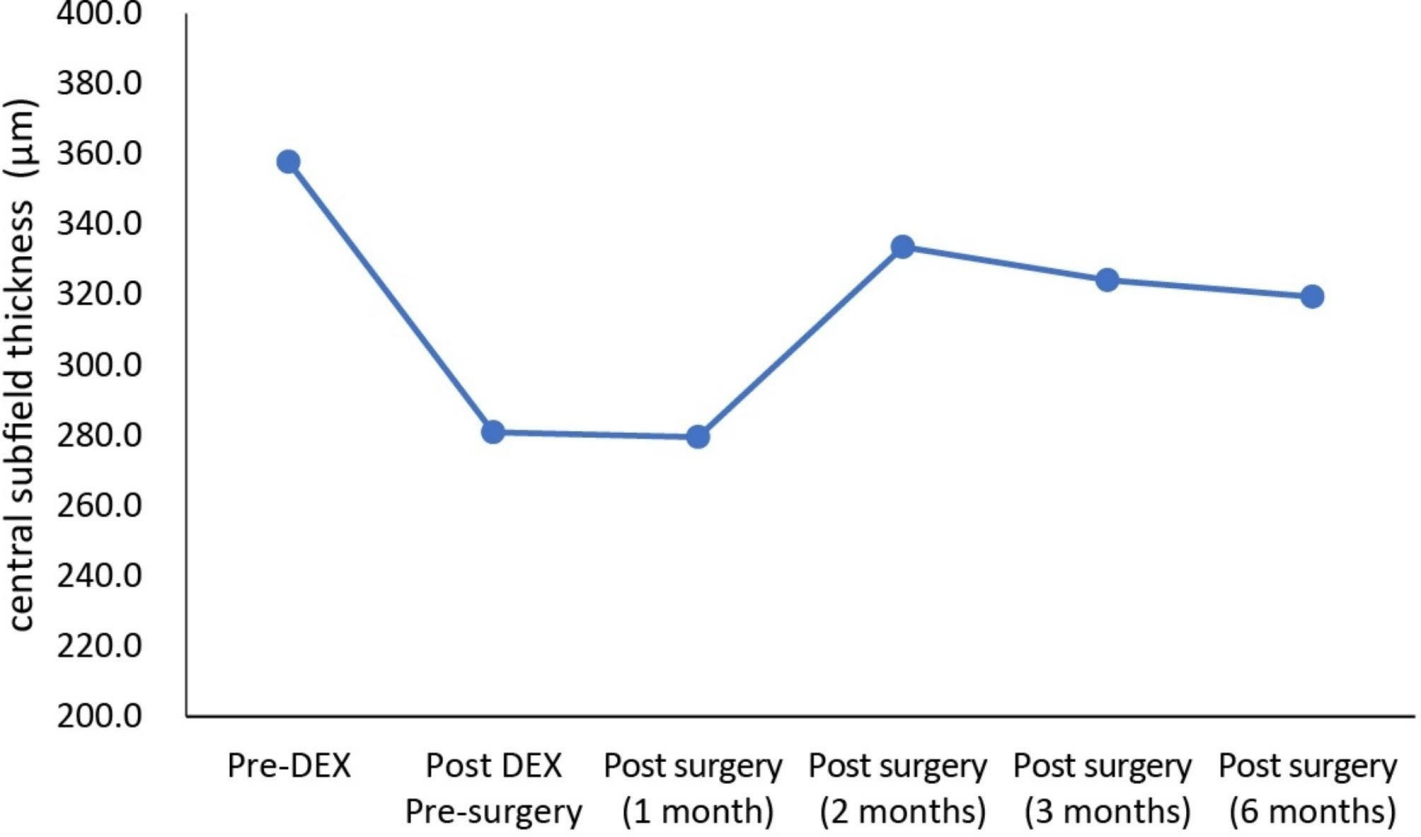
Central subfield thickness of the patients who received dexamethasone (DEX) implants followed by cataract surgery within 3 months. Pre-DEX: baseline injection of DEX implant last measurement (within one week), Post-DEX to surgery: last measurement after injection before cataract surgery (within one week), Post-surgery: after cataract surgery

At baseline (nearest measurement within one week of baseline injection of DEX implant), the mean CST for all eyes was 357.8 μm and decreased to 280.2 μm one week prior to cataract surgery and CST remained stable at 279.5 μm one month after cataract surgery (Fig. 1). The mean CST value remained below baseline at postoperative month 2 (333.6 μm), month 3 (324.2 μm) and month 6 (319.4 μm).

**Fig. 2:**
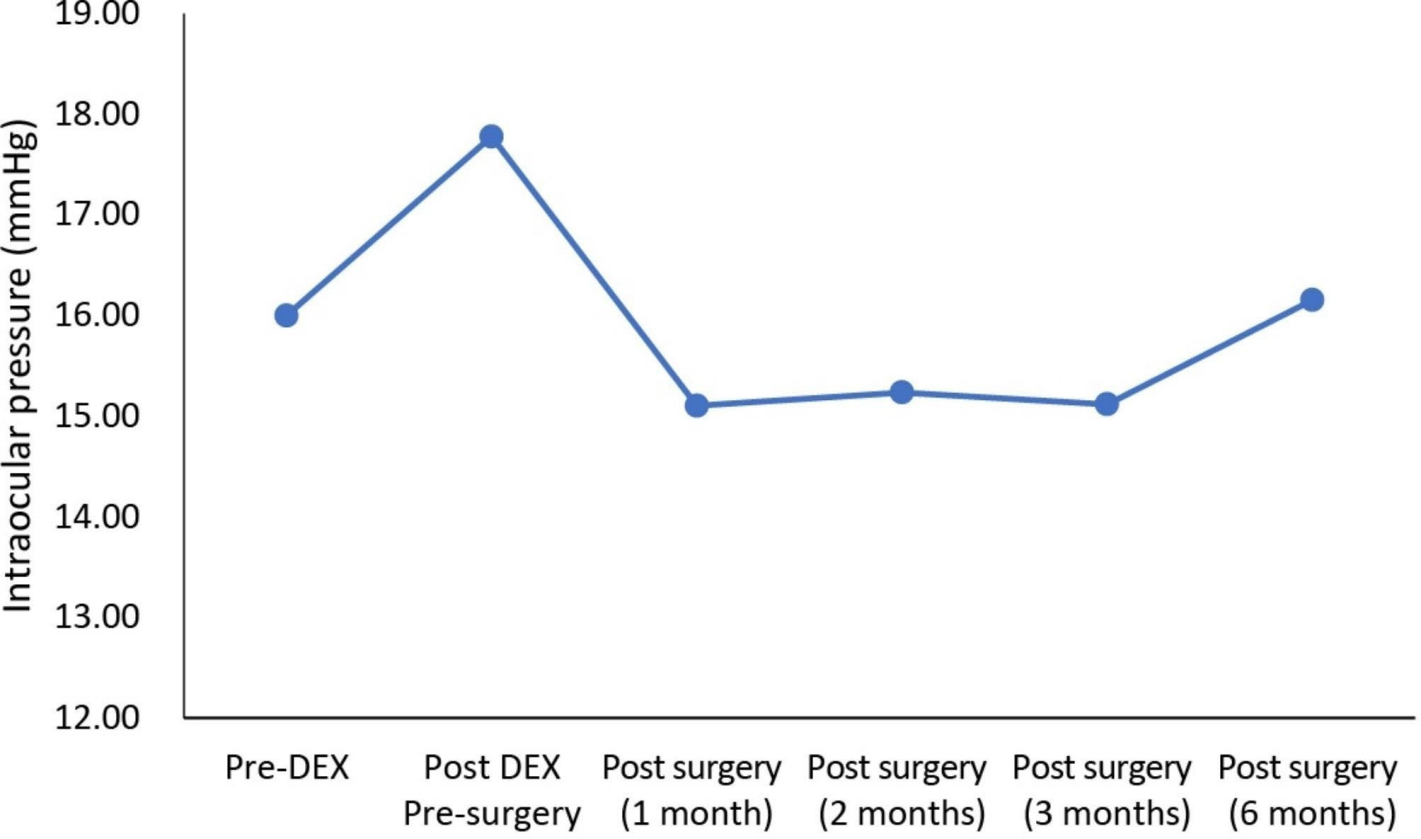
Intraocular pressure of the patients who received dexamethasone (DEX) implants followed by cataract surgery within 3 months. Pre-DEX: baseline injection of DEX implant last measurement (within one week), Post-DEX to surgery: last measurement after injection before cataract surgery (within one week), Post-surgery: after cataract surgery

At baseline, the mean IOP was 16.0 mmHg and increased to 17.8 mmHg before cataract surgery (Fig. 2). At month 1 after cataract surgery, IOP decreased below baseline to 15.1 mmHg. The IOP value was sustained at postoperative month 2 (15.2 mmHg) and month 3 (15.1 mm Hg) before returning to baseline at month 6 (16.2 mmHg). On average, no additional IOP-lowering drugs were needed during the follow-up visits at month 6 post-surgery.

**Fig. 3:**
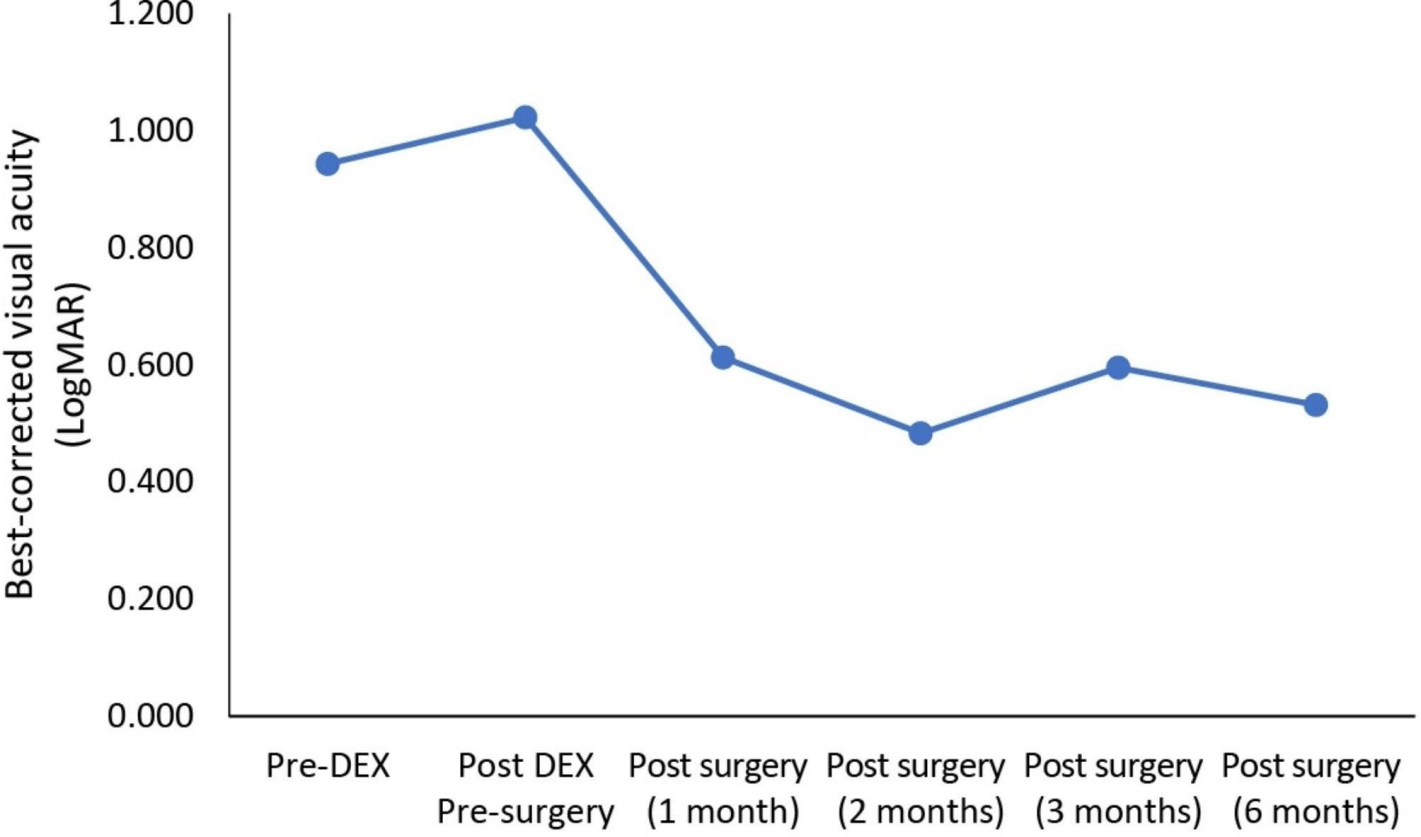
Best-corrected visual acuity (LogMAR) of the patients who received dexamethasone (DEX) implants followed by cataract surgery within 3 months. Pre-DEX: baseline injection of DEX implant last measurement (within one week), Post-DEX to surgery: last measurement after injection before cataract surgery (within one week), Post-surgery: after cataract surgery

The mean BCVA logMAR was 0.943 at baseline, before increasing to 1.022 before cataract surgery. After cataract surgery, BCVA logMAR improved to 0.613 at month 1, 0.483 at month 2, and 0.595 at month 3, and 0.532 at month 6 (Fig. 3). All patients had improvements in their vision.

## Discussion

Cataract surgery is one of the most common surgeries performed in developed countries [1]. Such surgery can complicate other conditions such as ME which occurs at a higher rate in both diabetic and RVO patients. ME has been demonstrated in prior studies to deteriorate after cataract surgery, and ME can arise in patients who have never had retinal impairment [17, 18]. In individuals with DME, an intravitreal DEX implant has been demonstrated to produce functional benefits comparable to anti-vascular endothelial growth factor (VEGF) medications but with greater anatomical responsiveness and fewer injections. In a published meta-analysis by He et al., intravitreal DEX implants showed improved anatomic outcomes over 6 months, with a mean CST decrease of around 87 μm when compared to anti-VEGF treatment [19]. The existing literature does indicate that approximately 30% of patients experience an increase in intraocular pressure following dexamethasone implantation. Similarly, our study findings demonstrated an average rise in intraocular pressure after the administration of Ozurdex prior to cataract surgery. However, it is noteworthy that after the surgical intervention, structural modifications and other unidentified factors (such as the potential reduction in intraocular pressure achieved through cataract removal in certain patients) contributed to maintaining the overall postoperative intraocular pressure close to the initial normal levels. Furthermore, the benefit of intravitreal DEX implant in DME peaked 4–8 weeks after insertion, making it a rational option in patients with both ME and cataracts, as it accommodates the time interval of putative postoperative inflammatory reaction while also assisting the resolution of pre-existing ME [14, 20].

As the percentage of people with diabetes is likely to rise in the coming years, the rate of diabetic individuals with cataracts is also projected to increase [21]. Clinical ME following small-incision phacoemulsification occurs in 0.1–2.35%, but it can be as high as 20% in diabetic patients with a history of DME treatment [22, 23]. Following cataract surgery, the normal course of ME in diabetic patients is yet unknown. The considerable variance in the occurrence of this exacerbation can be attributed, at least in part, to the diverse methodologies utilized to analyze postoperative ME deterioration based on whether the diagnosis was validated by optical coherence tomography, fluorescein angiography, or clinical examination alone. Inflammatory mediators appear to be the main trigger as they deteriorate the blood-retinal barrier, inducing elevated vascular permeability, pericytes, and endothelial cellular degeneration [24].

Intravitreal injections can efficiently deliver the prerequisite drug concentration when treating posterior eye disease. However, prior to the use of DEX implants, the short half-life of approximately 4 hours of DEX after a single intravitreal injection into the vitreous humor was a significant limitation [7]. DEX implants are micronized DEX-containing free-floating biodegradable copolymer composed of polylactic-co-glycolic acid. The copolymer breaks down into carbon dioxide and water over time, while DEX slowly diffuses into the vitreous cavity to impose its therapeutic benefit [25].

To develop an understanding of the thickness variations, the relationship of CST before surgery to that at 1 month, 2 months, 3 months, and 6 months following cataract surgery were studied. In this study, the mean CST value remained lower than the baseline at post 1 to 6 months of surgery (Fig. 1). The noted lower CST value indicated that the impact of DEX not only slowed the progression of edema caused by the inflammatory processes that occur during surgery and especially in the post-cataract period, but it also helped cure the edema as well [26]. Eight of these 19 eyes (42.1%) had a recurrence of ME during follow-ups, and all received a re-injection of DEX implant. The average interval between the re-injection and baseline injection was 122.4 days in these 8 patients. A DME recurrence rate of 21.6% (8/31 patients) has been reported previously for patients treated with DEX; however, these patients had not undergone surgery for cataracts [27]. A recurrence rate exceeding 90% has also been reported in patients by 180 days [28]. Similar injection intervals have been reported for chronic DME treated with DEX monotherapy, with Zandi et al. (2017) reporting an interval of 138 ± 15 days [29]. In another study, the injection interval for DME patients has been reported as 171 days (SD = 126) [30]. For patients with ME related to RVO, an interval of between 141 ± 33 days and 168 days has been reported in the literature [31, 32]. This suggests that the application of DEX implants prior to cataract surgery does not impact the rate of ME recurrence or the frequency of recurrence. Even though an interventional operation is required if the DEX implant is placed one month prior to surgery, improved postoperative inflammation control is achieved. Because implant insertion at the start or conclusion of the cataract treatment has possible risks, a safer implant injection was also assured. If the implant is placed at the start of cataract surgery, the surgeon may need to retrieve it if the posterior capsule ruptures as this would cause the implant to migrate into the anterior chamber where it can cause damage to the cornea [13]. Another concern is that the intravitreal injection would elevate the vitreous pressure, which may also increase the risks during cataract surgery. If the implant is introduced at the end of cataract surgery, however, there is an associated threat of potentially dangerous alterations to the globe and anterior chamber stability, which could jeopardize the procedure’s outcome [33].

Intravitreal corticosteroid injections are associated with increased IOP and cataract development. During the study period, none of the participants experienced an IOP greater than 22 mmHg, while normal IOP values range from 10 to 21 mmHg (15.5 mmHg ± 2 SD), and on average, no additional IOP-lowering drugs were needed. IOP did increase pre-surgery after baseline injection of DEX implant; however, this decreased post-surgery before returning to baseline level at month 6 post-surgery. A temporary increase in IOP was reported in the RVO and uveitis DEX implant phase III studies, usually peaking 60 days following DEX implant injection, and then only 24% of eyes sought therapy with topical IOP-lowering therapy. In contrast, intravitreal corticosteroid injections have been demonstrated to be associated with increased intraocular pressure (IOP) and cataract development [34]. The documented adverse event rates for cataract and cataract surgery after an average of five DEX implants for the treatment of diabetic ME (DME) during a three-year period were observed to be 67.9% and 59.2%, respectively [35].

Mean BCVA significantly improved in all the enrolled patients implying a better vision correction. A clinically meaningful rise in BCVA is considered to be a reduction of 0.3 logMAR [36]. In the present study, the averaged BCVA logMAR had improved from 0.943 at baseline to 0.532 6-months after cataract surgery. Previous research has found a slight correlation between CST decrease and improvement in BCVA, which could explain these observed findings [37–39]. In diabetic patients, clinical features such as advancing age, female gender, diabetes tenure, higher HbA1c level at the time of surgery, and moderately severe retinopathy have been linked to a poor prognosis following cataract surgery [40, 41]. These functional outcomes should be evaluated because, in the present study, a majority of the subjects were female with an increased mean HbA1c level prior to surgery.

The findings of this and earlier studies suggest that DEX implants could be a potential therapy for diabetic patients with post-surgical ME [16, 33, 36]. In real-world scenarios, it poses significant challenges to establish a control group consisting of patients with macular edema who receive cataract surgery without prior injection treatment. Such an approach would raise ethical concerns in terms of patient care. Hence, our objective in conducting this single-arm study was to compare our findings with existing relevant literature and draw meaningful conclusions. Our aim was to demonstrate the potential benefits of administering Ozurdex within three months prior to cataract surgery. Existing literature has consistently indicated that untreated macular edema, or even performing cataract surgery directly without prior injections, can exacerbate or perpetuate the condition of macular edema. By aligning our study’s conclusions with these existing findings, we anticipated shedding light on the positive impact of Ozurdex administration on the outcomes of cataract surgery. Despite differences in protocol layout, baseline variables, and goals among studies, evaluating the proportion necessary to treat based on the existing data allows for a minimal comparison of ME progression following cataract surgery. Before making a decision, the patient must be acquainted with all the complications and advantages of DEX implants. More research is essential to distinguish between diabetic people who are more likely to acquire ME and to validate the long-term effects and tolerability of DEX implants followed by a simple cataract surgery.

Given that the included eyes were limited (n = 19), we also suggest larger studies to replicate our findings with enough statistical power to detect outcome differences after DEX implants in patients receiving cataract surgery.

Finally, it’s essential to mention that the effectiveness of Ozurdex may vary over time, potentially impacting its anti-inflammatory effects following cataract surgery. However, due to practical reasons and individual patient factors, it may not always be feasible to promptly schedule cataract surgery within one month of Ozurdex administration. In our study, we investigated the outcomes of cataract surgery within three months after Ozurdex administration and found that the average CST remained below baseline values at postoperative months 2 (333.6 μm), 3 (324.2 μm), and 6 (319.4 μm). Additionally, all patients with ME showed visual improvements after cataract surgery following intravitreal DEX implants, suggesting potential benefits.

## Conclusion

Patients with ME due to diabetes or RVO may benefit from receiving DEX implants prior to cataract surgery within 3 months to achieve sufficient postoperative inflammation management and limit ME deterioration. In addition, patients with ME may benefit in vision gains from cataract surgery after prior intravitreal DEX implants. However, additional research will enable us to better comprehend the efficacy and safety of DEX implants in different patient demographics in the clinical context.

### Electronic supplementary material

Below is the link to the electronic supplementary material.

Supplementary Material 1

## Data Availability

The data used to support the findings of this study are available from the corresponding author upon request.

## Abbreviations

BCVA: Best-corrected visual acuity
CST: Central subfield thickness
DEX: Dexamethasone
DME: Diabetic macular edema
DR: Diabetic retinopathy
IOP: Intraocular pressure
ME: Macular edema
RVO: retinal vein occlusion
VEGF: Vascular endothelial growth factor
