# Supplementary figures and images for: Real-world experience on intravitreal dexamethasone implant in patients with macular edema scheduled to undergo cataract surgery
Source: BMC Ophthalmol. 2023 Aug 9;23:352. doi: 10.1186/s12886-023-03093-y (PMC10413593; doi:10.1186/s12886-023-03093-y)

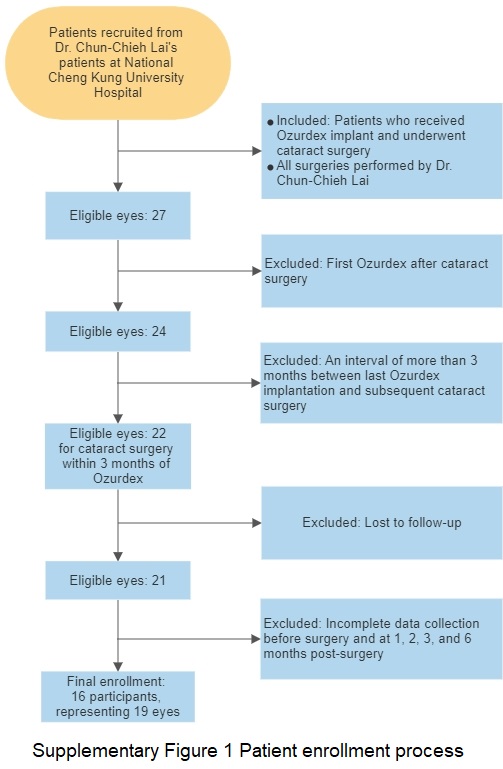

Supplement: Supplementary file 1 — Supplementary Material 1 [file 12886_2023_3093_MOESM1_ESM.jpg]
